# Antipsychotic prescribing choices in patients with First Episode Psychosis

**DOI:** 10.1192/j.eurpsy.2022.729

**Published:** 2022-09-01

**Authors:** J. Fallon, O. Tierney

**Affiliations:** 1 Brighton and Sussex Medical School, Medical Education, Brighton, United Kingdom; 2 Brighton and Sussex Medical School, Psychiatry, Brighton, United Kingdom

**Keywords:** schizophrénia, First Episode Psychosis, Prescribing, Antipsychotics

## Abstract

**Introduction:**

As all first line options in treating First Episode Psychosis (FEP) are similarly effective there is a consensus among prescribing guidelines that clinicians and patients should consider side-effect profile as the ‘driver’ of initial choice of antipsychotic. Anecdotally it has been observed that different care teams prescribe particular medications preferentially.

**Objectives:**

To evaluate the patterns of antipsychotic prescribing in patients with FEP at the time of initial treatment and over the first year with the Early Intervention Service (EIS).

**Methods:**

Medical records of all patients who had completed 1 year of follow-up with EIS in Sussex Partnership Foundation Trust (n=274) were reviewed. The first antipsychotic prescribed and antipsychotic prescribed at 12-months was recorded alongside initiating care team (EIS, non-EIS community services, inpatient services).

**Results:**

99% (n=272) of patients were prescribed an antipsychotic. 46% were initiated by inpatient serves, 40% non-EIS community services and 14% EIS. Aripiprazole, olanzapine, quetiapine and risperidone accounted for 95% of initial prescriptions. Different care teams prescribed antipsychotics preferentially (*p*=<0.005) **(Fig.1)**. Rates at which initial medication was continued at 12-months varied according to initial prescription (P=<0.05) **(Fig.2)**.

**Figure:**
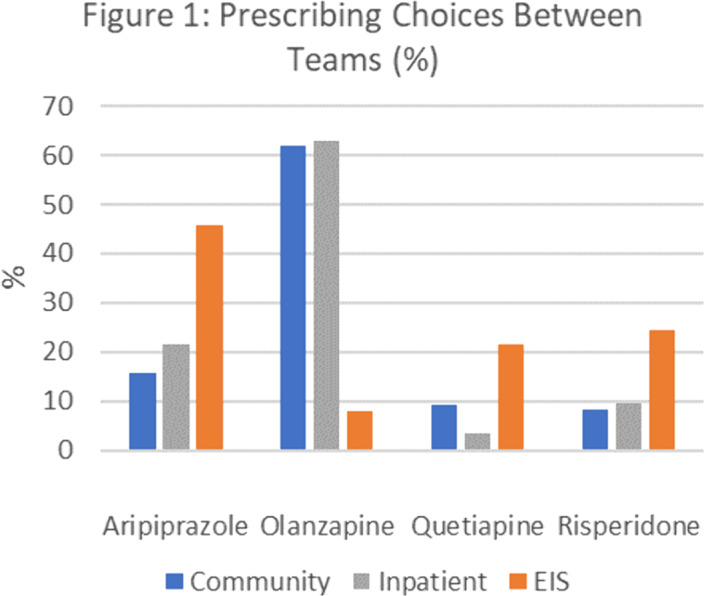

**Figure:**
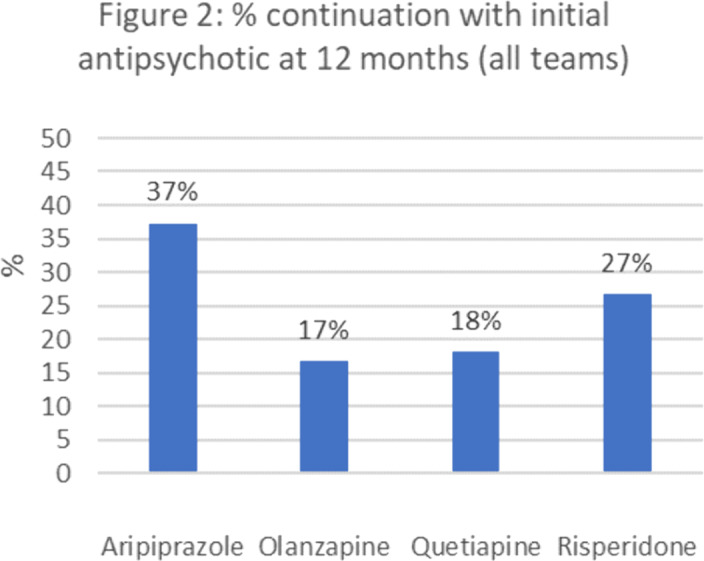

**Conclusions:**

The frequency that specialist EIS services prescribed aripiprazole as initial treatment contrasts the preference for olanzapine in other services. Olanzapine has a significant metabolic side effect profile, is sedating and was least likely to be continued at 12 months. This raises questions about why non-FEP specialist services prefer olanzapine and whether EIS services can support these services around initial medication choices more likely to be continued throughout the key first year of treatment.

**Disclosure:**

No significant relationships.

